# Intergenerational Interventions to Reduce Ageism: Roles and Outcomes of the Older Participants

**DOI:** 10.1093/geroni/igaf122.3797

**Published:** 2025-12-31

**Authors:** Brian Carpenter, Sizhe Li, Claire Wininger, Natalie Galucia, Aaron Li, Nancy Morrow-Howell

**Affiliations:** Washington University, St. Louis, Missouri, United States; Washington University, Saint Louis, Missouri, United States; Washington University, Saint Louis, Missouri, United States; Washington University, Saint Louis, Missouri, United States; Washington University, St. Louis, Missouri, United States; Washington University, St. Louis, Missouri, United States

## Abstract

Intergenerational interventions to reduce ageism have focused primarily on the important aim of changing attitudes among younger people. Older adults are included as essential components of interventions, but they are rarely viewed as targets of change or beneficiaries themselves. In the current analysis, we synthesize literature to understand how older adults are included in anti-ageism interventions. We build from a systematic review of interventions by Apriceno and Levy (2023) that summarized intervention effectiveness. We analyzed these articles for the complementary purpose of identifying roles of older adults in interventions. Of 123 articles in the systematic review, 88 included interventions involving personal contact with older persons. In those 88 interventions, older participants were mostly female and Caucasian, with a wide age range, and they were recruited primarily from residential facilities (n = 30 interventions, 34%) and via general community outreach (n = 28, 32%). The most common activities in the interventions were guided conversations/discussions (n = 52, 59%) and creative activities (n = 21, 24%). Only 13 of those 88 programs (15%) evaluated older adults directly and reported outcomes for them. Outcomes focused on attitudes toward younger people, self-esteem, life satisfaction, and generativity and were generally positive, particularly for attitude shifts. However, even when studies did evaluate older adults, they had substantial methodological weaknesses that limit rigorous hypothesis testing and generalizability. In sum, older adults receive cursory attention in intergenerational interventions to combat ageism, despite their central role. Future studies should integrate older adults more systematically into anti-ageism interventions.

